# Investigating the benefits of artificial neural networks over linear approaches to BMI decoding

**DOI:** 10.1088/1741-2552/ade568

**Published:** 2025-06-27

**Authors:** Hisham Temmar, Matthew S Willsey, Joseph T Costello, Matthew J Mender, Luis Hernan Cubillos, Jesse C DeMatteo, Jordan LW Lam, Dylan M Wallace, Madison M Kelberman, Parag G Patil, Cynthia A Chestek

**Affiliations:** 1 Department of Biomedical Engineering, University of Michigan, Ann Arbor, MI, United States of America; 2 Department of Neurosurgery, University of Michigan Medical School, Ann Arbor, MI, United States of America; 3 Departments of Electrical Engineering and Computer Science, University of Michigan, Ann Arbor, MI, United States of America; 4 Department of Robotics, University of Michigan, Ann Arbor, MI, United States of America

**Keywords:** brain–machine interface, nonlinear decoding, neural networks, non-human primate, intracortical, motor cortex

## Abstract

*Objective.* Brain–machine interfaces (BMI) aim to restore function to persons living with spinal cord injuries by ‘decoding’ neural signals into behavior. Recently, nonlinear BMI decoders have outperformed previous state-of-the-art linear decoders, but few studies have investigated what specific improvements these nonlinear approaches provide. In this study, we compare how nonlinear and linear approaches predict individuated finger movements in open and closed-loop settings. *Approach.* Two adult male rhesus macaques were implanted with Utah arrays in the motor cortex and performed a 2D dexterous finger movement task for a juice reward. Multiple linear and nonlinear ‘decoders’ were used to map from recorded spiking band power into movement kinematics. Performance of these decoders was compared and analyzed to determine how nonlinear decoders perform in both open and closed-loop scenarios. *Main Results.* We show that nonlinear decoders enable control which more closely resembles true hand movements, producing distributions of velocities 80.7% closer to true hand control than linear decoders. Addressing concerns that neural networks may come to inconsistent solutions, we find that regularization techniques improve the consistency of temporally-convolved feedforward neural network convergence by up to 188.9%, along with improving average performance and training speed. Finally, we show that TCNs and long short-term memory can effectively leverage training data from multiple task variations to improve generalization. *Significance.* The results of this study support artificial neural networks of all kinds as the future of BMI decoding and show potential for generalizing over less constrained tasks.

## 1. Introduction

When asked about priorities for recovery using various neurotechnologies, people living with tetraplegia have consistently held restoration of upper-limb fine motor skills at the top [1–3]. Over the past few decades, researchers have pursued this goal through the development of brain–machine interfaces (BMIs), which record neural activity through microelectrodes implanted in the brain and use a ‘decoder’ to map this activity to a control signal for prostheses. Using BMIs, human study participants have successfully produced text and speech [4–6], controlled computer cursors [7, 8] robotic arms [9, 10], prosthetic fingers [11], and, through muscle stimulation, controlled an individual’s own arm [12, 13].

For the better part of the last two decades, linear decoders have dominated the state of the art in the BMI field [14–19]. These approaches were motivated by studies showing apparent linear relationships between single-neuron firing rates and behavioral variables like kinematics of a reaching task or muscle activity of a precision grip [20–24]. However, more recent work has suggested that the true relationship is more complicated: neural activity is driven at a population level by latent ‘neural trajectories’ through lower dimensional neural subspaces/manifolds, and these trajectories may have a highly nonlinear relationship to behavior [25–31]. While linear decoders may be especially capable of predicting multi-DOF single-effector movements in constrained settings, recent studies have observed that linear decoders tested outside of their specific task may break down [29, 32]. As tasks become more sophisticated, the field had looked to nonlinear decoders as a potential solution for high-performance BMIs.

As a result, nonlinear models have now been used to significantly outperform linear methods in both offline and real-time BMI decoding tasks [4, 5, 33–36]. In particular, nonlinear BMIs have recently enabled high-performance speech decoding in real-time [6, 37]. Recently, our group developed both a temporally-convolved feedforward neural network **(TCN)** and long-short term recurrent neural network (LSTM) which outperformed previous state-of-the-art decoders like the ReFIT Kalman Filter (RKF) in a 2-degree-of-freedom (DOF) dexterous finger movement task [38, 39].

**Figure 1.**
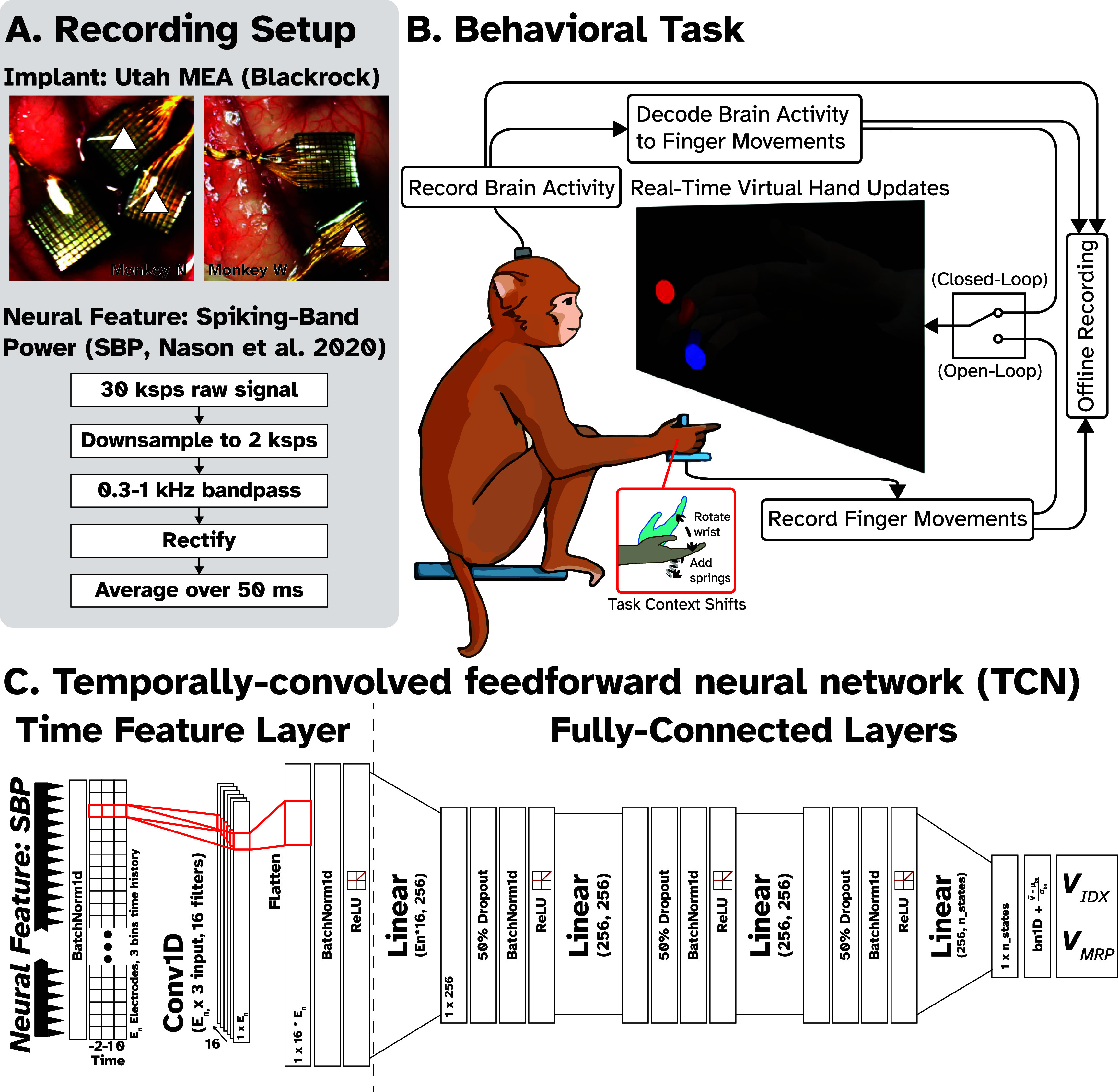
Experimental setup for finger movement BMI experiments. (A) *Top:* Utah arrays implanted in Monkey N (left) and Monkey W (right). The white triangles indicate which arrays were used for decoding. In Monkey N, the two arrays marked with a white triangle are 8 × 8 Utah arrays connected to the same pedestal in the motor cortex (precentral gyrus). In Monkey W, we used the lateral of two 96-channel arrays also implanted in motor cortex. *Bottom*: Flowchart describing neural feature extraction. 96 channels of 30 ksps raw data are down-sampled, filtered, rectified, and averaged, resulting in a low-power signal containing spiking information, called Spiking-Band Power (SBP, Nason *et al* 2020) [40]. (B) During an experiment, the monkey must move a virtual hand to match targets presented as spheres, 1 for each degree-of-freedom (DOF). During hand control (open-loop) trials, this is accomplished by moving the fingers in the manipulandum to match the same posture. The manipulandum measures finger flexion using flex sensors. In closed-loop (online) trials, the virtual hand is controlled by the output of the neural decoder, which maps brain activity to finger velocities. Additionally, for some trials, we introduced two ‘context shifts’ to the task, where either torsion springs were added to the flexion joint of the manipulandum to increase flexion force or the wrist angle was changed. (C) The primary decoder used in this study is a **T**emporally **C**onvolved feedforward neural **N**etwork (**TCN**), whose architecture is outlined above.

While the use of artificial neural network (ANN) decoders has significantly improved BMI performance in tightly controlled experimental settings, it is unclear how well they will generalize to real-world use cases. As neural activity has been shown to shift significantly between variations to a task, it is possible that ANNs may be sacrificing robustness to achieve such high-performance [32]. A recent study demonstrated a recurrent neural network being effectively used to control two cursors simultaneously in real time, but it also demonstrated that without proper training, models may overfit to training datasets and fail during closed-loop control [36]. Additionally, stochasticity in ANN training approaches may lead to model instability, or variability in training, even when trained on the same data [39]. Although an increasing number of studies have focused on adapting known ML architectures to neural decoding, there is a need to better understand whether ANNs will generalize and consistently converge to high-performing decoders. The focus of this study is not to introduce a new state-of-the-art decoder, but to examine how multiple nonlinear decoders outperform linear methods, as well as evaluate some potential points of failure these decoders may encounter. These include, for example, how consistently they can converge on similar solutions, and the potential impacts of using randomly seeded nonlinear decoders on generalization to unseen tasks. To this end, we evaluated the performance of multiple nonlinear decoders compared to basic linear methods, focusing mainly on the offline and online performance of a TCN whose architecture is shown in figure 1(C).

In this study, we show that offline, three nonlinear decoders (including TCN) outperform a linear decoder by producing a range of velocities that more closely match those of natural hand finger movements. In addition, during real-time experiments, TCN and its recalibrated real-time counterpart, ReFIT-TCN, maintain these improvements over the previous state-of-the-art linear approach (the RKF). We then find that regularization techniques developed within the last decade enable TCN models to converge on high-performance solutions quickly and consistently across training runs, both offline and online. We also find that any remaining variance in TCN convergence is negligible during real-time control. Finally, we demonstrate that linear, TCN, and LSTM (long short-term memory recurrent neural network) decoders overfit to training data and struggle to generalize to task variations (‘contexts’). However, by including data from multiple contexts, this overfitting can be mitigated, and decoders can achieve near-indistinguishable performance from decoders trained and tested in the same context. We believe that these findings will allow researchers to improve the performance of their ANN decoders, regardless of their core architecture (e.g. RNN, convolutional neural network (CNN), transformer), as well as improving their generalization for real-world use.

## 2. Materials and methods

### 2.1. Experimental design

This study investigated the ability of nonlinear decoders to outperform current linear methods as well as to investigate the potential of ANN decoders to converge consistently and generalize to task context. The experiments were designed to collect synchronous brain and behavioral data during a two DOF finger movement task, as well as to test the performance of BMI decoders on said task. This study includes data from two non-human primates, and no power analysis was calculated before the study. The protocols in this study were approved by the Institutional Animal Care and Use Committee at the University of Michigan.

### 2.2. Implants

Two non-human primates, Monkey N and Monkey W (NHP, Rhesus macaque, *Macaca mulatta*) was implanted with Utah microelectrode arrays (Blackrock Neurotech, Salt Lake City, UT) in the hand area of the precentral gyrus, as described previously [16, 32, 41, 42]. Monkey N was implanted with two 64-channel arrays in the right hemisphere of motor cortex (precentral gyrus), marked with two triangles in figure 1(A). Monkey W was implanted with two 96-channel arrays in the right hemisphere of the motor cortex (precentral gyrus), seen in figure 1(A). Simultaneous recording from multiple channels was limited to 96 channels. In real-time decoding channels with low activity (<1 Hz spiking rate) were typically excluded from real-time decoding, as were channels with visible noise. However, all 96 channels were used in offline analyses. In this study, recordings from Monkey N were captured between 578 and 1519 d post-cortical implant. Recordings from Monkey W were captured between 81 and 224 d post-cortical implant.

### 2.3. Decoding hardware and neural feature extraction

Signals from the implanted Utah array were recorded in real time through the Cerebus Neural Signal Processor (Blackrock Neurotech, Salt Lake City, UT). The BMI is an xPC Target (Mathworks, Natlick, MA) based system. In this study, we used spiking-band power (SBP) as our neural feature, where raw 30 ksps data is downsampled to 2 ksps, a 300–1000 Hz bandpass filter is applied, and the resulting value is rectified, as described previously [40, 43]. This 2 ksps SBP feature is then further averaged over 50 ms into non-overlapping bins for decoder training and testing. In real-time experiments, binned SBP data was sent to a separate Linux machine which decoded the neural data and sent back the predicted kinematics.

### 2.4. Behavioral task

Both Monkeys N and W were trained to sit in a chair (Crist Instruments, Hagerstown, MA) and perform a dexterous finger movement task. During the task, each monkey was presented with a monitor showing a virtual hand, which was controlled either by their physical hand in a manipulandum or by using decoded neural activity (figure 1(B)). In hand-control mode (termed ‘HC’), moving the index (IDX) or middle-ring-small (MRS) finger groups controlled the corresponding virtual finger groups. Offline analyses refer to predicting the finger kinematics from the neural activity using HC. In brain-control mode (‘online’ control), neural activity was decoded to predict virtual hand movement in real-time. Hand movement data was captured using flex sensors placed in a manipulandum in which the monkeys’ hands were placed (FS-L-0073-103-ST, Spectra Symbol, Salt Lake City, UT). The actual task performed by the monkeys is to match pseudo-randomly presented targets for each finger group (IDX or MRS), as described in previous studies [16, 38]. The monkeys received a juice reward for successful trials. Hold times for successful trial completion were 750 ms in ‘training’ mode and 500 ms in ‘testing’ mode. The failure condition was a trial timeout of 10 s, except for days 1449, 1456, and 1463 post-implant in Monkey N, which had a timeout of 5 s, days 81 and 98 for Monkey W, which had a timeout of 7.5 s, and day 224 for Monkey W, which had a timeout of 20 s. Target size was 15% of the movement range. Positions for each DOF will be referred to either as %flexion or %extension, and velocity units are in flex s^−1^, where ‘flex’ is the proportion of the total movement range from 0 (full extension) to 1 (full flexion) for either degree of freedom. This means that positive velocities are flexion and negative velocities are extension.

On some days, we shifted the ‘context’ of the task in one of three ways: (i) by adding springs to the manipulandum to increase the required flexion force, (ii) by moving the wrist 23° into flexion, or (iii) by applying both shifts at the same time. These shifts have previously been shown to modulate neural activity while maintaining similar kinematics [32]. Applying these shifts results in four ‘task contexts’: the *spring* context, the *wrist* context, *spring-wrist* context (both simultaneously), and *normal* context (no shifts).

### 2.5. Datasets

Below, we describe the datasets used in analyses throughout the study. Additionally, table 1 reports the date, post-implant day, monkey, and a brief description of each session used in the study.

**Table 1.** List of datasets. Each row indicates an experimental session used in the study. Columns show the date of the session, the number of days post implant, a short description indicating what kind of experiment was performed. These are further described in the Datasets section of the Methods and Materials. In the details for the Offline Four-Context sessions, single letter abbreviations indicate the order in which the blocks of context trials were performed (N = Normal, W = Wrist, SW = Spring-Wrist, S = Spring).

Monkey	Date (YYYY-MM-DD)	Days post-implant	Additional details
N	2021-02-16	735	Offline Random Target Acquisitions
	2021-04-12	790	Offline Random Target Acquisitions
	2022-06-16	1220	Offline Random Target Acquisitions
	2022-09-06	1302	Offline Random Target Acquisitions
	2022-05-31	1204	Offline Four-Contexts: N, W, SW, S
	2022-06-02	1206	Offline Four-Contexts: N, S, SW, W
	2023-01-17	1435	Offline Four-Contexts: N, SW, S, W
	2023-04-07	1515	Offline Four-Contexts: S, SW, N, W
	2023-04-11	1519	Offline Four-Contexts: SW, W, N, S
	2020-09-12	577	RTCN/RKF ABAB comparisons
	2020-09-19	584	RTCN/RKF ABAB comparisons
	2021-07-31	899	RTCN/RKF ABAB comparisons
	2022-02-02	1086	Real-time TCN variance comparison, 5 TCNs
	2023-01-31	1449	Real-time TCN variance comparison, 5 TCNs
	2023-02-07	1456	Real-time TCN variance comparison, 1 TCN
	2023-02-14	1463	Real-time TCN variance comparison, 1 TCN

W	2020-11-21	81	TCN/RTCN AB comparisons
	2020-12-08	98	RKF/TCN ABA comparisons
	2020-12-23	113	TCN/RTCN AB comparisons
	2021-04-14	224	Offline Random Target Acquisitions

*Offline random target acquisitions:* on four days, Monkey N performed 1000+ trials of the random target finger movement task. On one day, Monkey W performed 750+ trials of the random target movement task. Data for each day was split into training and test datasets for offline decoding according to the leave-one-out approach described in Offline Decoding Analyses.

*Offline four-context random targets:* on five days, Monkey N performed 600+ trials of the random target task for each of the four contexts previously described to evaluate decoder performance across contexts. Data for each context (and within each day) was split into five 400 trial training datasets and one 100-trial testing dataset, according to the leave-one-out approach described in Offline Decoding Analyses. Additionally, we prepared two types of training datasets with multi-context data, ‘full’ and ‘mixed’-context, as described in Offline Decoding Analyses, resulting in 10 mixed context datasets per day.

*Real-time decoder comparisons: TCN/RKF ABAB comparisons:* For Monkey N, ReFIT TCN (RTCN) and the RKF were compared in ABAB comparisons on three days. For Monkey W, due to inconsistent behavior, RTCN and RKF were not compared within the same day, so three comparisons on separate days, TCN vs. RTCN, RKF vs. TCN, and TCN vs. RTCN, are included. Additionally, offline training data from these three sessions were used for offline decoder training and testing, as described in Offline Decoding Analyses. Details on all these comparisons can be found in the 2022 study by Willsey *et al* [38].

*Real-time TCN variance comparisons:* four total days of online control experiments were performed for this dataset, with the goal of evaluating the variance in performance of online decoders trained on identical data. On 2 d we trained 5 TCN decoders on a 400-trial random target dataset (within each day) and tested them in successive 100+ trial blocks. On 2 additional days, we similarly trained a single TCN decoder and tested it in five 100+ trial blocks. Since the stochastic initialization of TCN parameters could contribute to variance in decoder performance, we chose not to apply the ReFIT procedure for these real-time comparisons, therefore the decoders used on these days are TCN, not RTCN.

### 2.6. Decoders

Throughout this study, we compare the offline predictions and real-time performance of multiple linear and nonlinear approaches to decoding. Each of these approaches is described in more detail below. Offline trials are also referred to as ‘hand-control’ (HC) trials. In this study, we use the words ‘model’ and ‘decoder’ interchangeably. While we use RR and TCN in our offline analyses, online, we opted to use higher performance analogs in our online comparisons, RKF and ReFIT-TCN (RTCN) [18, 38, 44].

*Ridge Regression (RR)* [45]: RR was used as a representative example of an offline linear decoder. While similar to linear regression, RR includes L2 regularization, which aims to balance model performance with the magnitude of model weights, as determined by a hyperparameter lambda. A lambda value of .001 was used, determined by performing a hyperparameter sweep over preliminary data.

*RKF* [18]: Kalman filters makes use of a linear dynamical model to incorporate prior knowledge of estimated behavior and the current observation to predict the true value of a hidden state, such as the velocity of two finger groups controlled by a BMI. The RKF improves on the basic Kalman filter by implementing a recalibration stage in real-time control to improve performance. The RKF implementation used in this study is described in Nason *et al* 2021 [16].

*Dual State Decoder (DS)* [34]: Sachs *et al* 2016 proposed a DS decoding paradigm, which predicts movement velocities by taking a weighted linear combination of two linear regressions trained on either high-speed movements or low-speed movements (referred to as the ‘movement’ and ‘posture’ states in the original study). The weights are determined by a modified LDA classifier, which is trained to predict the likelihood of being in either the movement or posture state from the same neural data used in the linear regression. The classifier also features an adaptive threshold, which aims to maintain a pre-defined ratio by measuring the proportion of fast and slow classifications over a sliding window and adjusting accordingly. In this study, we chose a ratio of 1:1.

*Temporally Convolved Feedforward Neural Network (TCN)* [38]: The TCN network, whose architecture is outlined in figure 1(C), was first proposed by Willsey *et al* in 2022 [38]. The same hyperparameters were used as in the previous publication. At the input, the network applies multiple time-wise convolutions across 3 time-steps of the neural feature to each channel independently, unlike a ‘traditional’ CNN, where the filter bank is iterated over parts of an entire signal (like passing over an entire image). The TCN was implemented using PyTorch with standard linear, batchnorm, and convolutional layers. Convolutional filters were trained on 3-bins of time history (150 ms total).

*ReFIT Temporally Convolved Feedforward Neural Network (RTCN)* [38]: The RTCN is a variant of the TCN which is trained using a similar recalibration stage as the RKF. Briefly, after an initial online session, recorded online velocities pointed away from the target are ‘corrected’ to produce a ‘refit’ training data set. The original TCN is then fine-tuned on the ‘refit’ data for 500 iterations at a learning rate of 2 × 10^−4^.

*Non-regularized TCN:* We also trained versions of TCN without batch normalization (BN) and artificial neuron dropout (DP), two training strategies which have empirically been shown to help ANNs regularize [46, 47]. Otherwise, the same architecture, initialization, and optimization were used. These variants are called TCN_noBNDP (no DP or BN), TCN_onlyBN (BN only), and TCN_onlyDP (DP only). Together, these models are referred to as the ‘ablated’ TCN models. We did not perform additional hyperparameter optimization on the ablated models.

*LSTM Recurrent Neural Network:* the LSTM used in this work had the same architecture and was trained in the same fashion as the LSTM in Costello *et al* in 2023 [39]. LSTM was implemented using the torch.nn.LSTM class, with one layer and a hidden size of 300. During training, each sample had a sequence length of 20 and zero-initialization for the hidden state. The final output of each sample was used to compute loss. LSTMs were trained using the Adam optimizer, with an initial learning rate of 2 × 10^−4^, weight decay of 0.003, and a plateau learning rate scheduler (800 iteration patience, 2 steps, factor of 0.5). Additionally, SBP and behavior were both normalized during training was normalized and two types of noise were added to each channel per batch: a bias sampled from a zero-mean Gaussian distribution with 0.1 standard deviation, and white noise to each channel and time point sampled from a zero-mean and 0.2 standard deviation.

*Additional notes on TCN architecture:* All neural networks were trained in PyTorch to minimize MSE between predicted and true velocities using the Adam optimizer (learning rate 1 × 10^−4^, weight decay 1 × 10^−2^). While networks intended for closed-loop experiments were trained for 3500 iterations with triangular velocity redistribution, offline networks were trained for 10 epochs with no velocity redistribution, with a few exceptions: The TCN_noBNDP and TCN_onlyDP ablated models, which were trained for 80 epochs each, and all models trained on normalized neural and behavioral activity were trained for 15 epochs each. Additionally, TCN normalizes the final velocity output, which is then scaled back to true velocity ranges. In the 2022 study, this was performed by using the median velocity as an offset and scaling to training velocity peaks. In this study, besides the three days of RTCN/TCN/RKF comparisons, normalized velocities are instead scaled by using the training data to train two independent RRs (each with a single weight, single bias) to map from the normalized IDX/MRS predictions to the final IDX/MRS predictions.

*Time history:* For all decoders except for RKF, 100 ms (2 bins) of lookback (time history) were also included for each SBP feature. This means that each decoder was trained to predict velocities from 150 ms (3 bins) of averaged SBP on each channel.

### 2.7. Offline decoding analyses

*Offline data preparation:* on each day of offline trials, unsuccessful trials were removed, and the dataset was trimmed to the middle 600 trials. The final 100 trials in the 600-trial block were then held out and not seen by decoders until testing. The first 500-trial dataset was randomly shuffled and split into five equal length blocks. Then, using a five-fold approach, data was split into five ∼400 trial training datasets (a.k.a. training folds) for each day. The remaining 100 trials in each fold were used as validation but were not included in testing datasets. Since Monkey W only had a single session of extended offline target acquisitions, we also prepared the offline training data from the three online comparison days in a similar fashion, leaving the final 100 trials for testing and splitting the remaining 300 into five 240 trials training datasets for each day.

*Multi-context data preparation:* on days where multiple contexts were tested, the same data preparation procedure was followed within each context, producing five 400-trial training datasets and one 100-trial testing dataset per context. Furthermore, within each fold, we constructed two datasets containing examples of all four contexts, called ‘full’ and ‘mixed’-context. The full-context datasets contain all the training data from each context within a fold, while the ‘mixed’-context datasets contain only 25% of the data from each context. Since training data was already randomly shuffled to remove time effects, the data from each context in the mixed datasets was sampled sequentially, i.e., the first 25% of the first context, the next 25% of the second context, etc.). This resulted in five 1600-trial ‘full’ datasets and five 400-trial ‘mixed’ datasets.

*Offline error:* offline prediction error was measured using the mean-squared error (MSE) between decoder predictions and true velocities. Since most analyses include predictions from multiple instances of similar decoders, MSEs are typically reported as the average MSE across all decoders of the same type and are accompanied with a standard error.

*Comparing speed regimes:* we compared the mean speeds and MSEs of offline decoder predictions in two ‘speed regimes’, high- and low-speed. Here, speed refers to the velocity magnitude within a single degree of freedom. We defined the high-speed and low-speed regimes within a day, as the time points containing top and bottom 10th percentiles of true speeds (across both degrees of freedom), respectively. This allowed us to investigate both how decoders differ at high and low speeds and if these differences are closer to true hand movements.

*Comparing distribution of predicted velocities:* we measured the Kullback–Leibler divergence (KL-div) between the distribution true velocities and velocities generated by a decoder. PMFs were estimated by scaling histograms of velocities (across DOFs) to sum to one. When comparing the distributions of decoders tested in real-time, we used the histogram of the experimental day’s training data to calculate the histogram.

*Median prediction deviation:* We tested if the ablated (TCN_onlyDP, TCN_onlyBN, TCN_noBNDP) models converged less consistently on the solutions than TCN within a day. For each velocity measurement in the test dataset, we measured the standard deviation of velocity predictions across all instances of each model type. We took the median of the resulting N × 2 vector across all time points and degrees of freedom, resulting in a scalar measure of how much each model type varied in convergence per day. This was further averaged over days.

*Context comparisons:* on days with trials performed in different contexts, decoders were trained for each single- and mixed-context dataset. All decoders from one day were then tested on the four single-context testing datasets for that day. MSE of these predictions were then grouped by three prediction types, *on-context* (training context matches test context), *off-context* (training context does not match test context), and *mixed-context* (training contains multiple contexts).

### 2.8. Online decoding

*Online data preparation:* For all analysis of online trials, the first 5 trials of any contiguous block of online trials were excluded to allow Monkey N to become familiar with each decoder. Additionally, only the middle 100 trials of each block were included for analyses on the real-time TCN variance experiments, or all trials if there were <100 (lowest was 95). For sessions comparing two decoders (RKF, RTCN, or TCN), all successful, closed-loop trials where the monkey was performing the task were included.

In an initial real-time TCN variance experiment, we observed that Monkey N progressively improved over the course of a session. To reduce the influence of behavior factors in following sessions, we had Monkey N perform an additional trial block at the end of each experiment using the first decoder tested, not included in analyses. For the single decoder variance experiments, this meant running 6 blocks of the same decoder. If there was a large difference in performance between trials performed in the first and last blocks of the session, we would exclude the day, however no days needed to be excluded except the initial experiment.

*Performance metrics:* general online performance was assessed using throughput as defined by Fitt’s law, measured in bits per second. Time-to-target (TT) was used to assess a decoder’s ability to quickly reach the target, and was measured as the time from the start of a trial to the first time both finger groups entered their respective targets, as described previously [16]. Orbiting time (OT) was used to assess a decoder’s ability to correct overshoots once targets were reached and was measured as the time from the first target acquisition (the endpoint of TT) to the final time the targets are acquired by both finger groups before the hold time is completed, as described previously [16]. Orbiting Rate (OR) was used to assess a decoder’s ability to prevent overshooting and was measured as the ratio of trials with nonzero OT to total trials performed by the decoder. In analyses investigating trial orbiting, both OR and non-zero OT are used.

### 2.9. Statistical analyses

In general, all statistical comparisons between decoder performances online were performed by grouping all decoders of each type across all days and performing a two-sample, one-tailed *t*-test with an alpha level of 0.01, unless otherwise noted here. When comparing ORs between RTCN and the RKD, a difference of proportions test (across days) was used. To compare the variance of multiple decoders trained on the same data online, we used a nested two-way ANOVA, with the two factors being the session number (date) and the run within the session (on the days with multiple decoders, each run is a different decoder). In offline scenarios, paired *t*-tests were used where appropriate, since many comparisons were performed between instances of different decoders that were trained on the same data and then tested on the same holdout. However, a two-sample *t*-test was used for comparing average offline performances between TCN and ablated versions.

## 3. Results

### 3.1. Improvements to the ‘fit’ of offline predictions using nonlinear decoders

**Figure 2.**
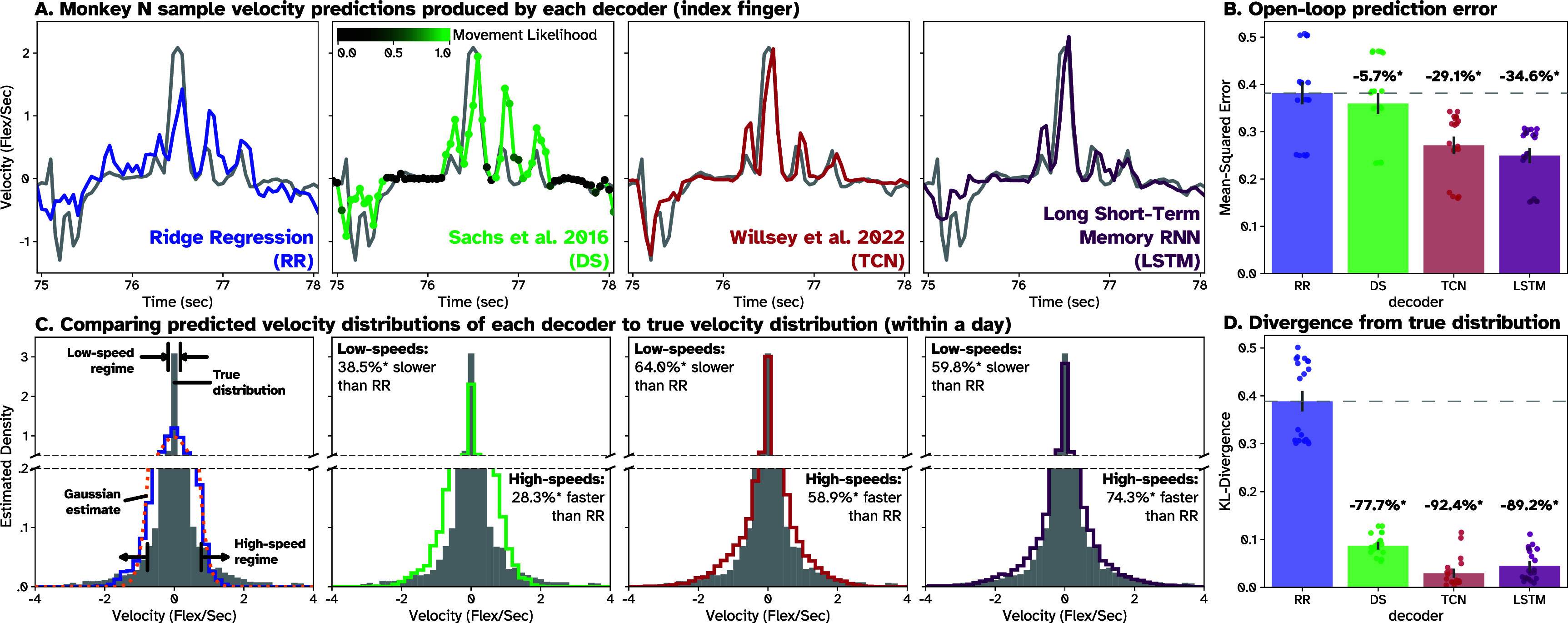
Distribution of nonlinear decoder predictions in Monkey N better matches hand control. The four decoders compared here are Ridge Regression with three time bins of history (RR, blue), a dual-state movement-posture decoder proposed by Sachs *et al* in 2016 [34] (DS, green), TCN (red), and a long-short term memory recurrent neural network (LSTM). True hand control (HC) is also shown in gray in subfigures (A) and (C). (A) Sample predictions from each decoder on the same movement. (B) Average MSE of open-loop decoder predictions across four days on a 100-trial holdout set within each day. Gray lines show the standard error about each mean MSE. Asterisks (*) above a bar indicates *p* < .01 when comparing average MSE to RR, with the average % difference from RR across all decoders and days reported as well. (C) Example histograms of the distribution of velocities produced by actual hand movements in gray and by each decoder in their respective colors. We observed that the RR distribution appeared somewhat Gaussian, so for each session we used the RR predictions to estimate the mean and SD of a normal distribution. The distribution for one session is included in the leftmost plot (orange dashed line). Broken axes are used to highlight estimated density in high and low speed regimes. The percentage differences in the center/right panels are calculated by taking the percentage difference in mean speed at high and low speed regimes between RR and DS/TCN/LSTM and then averaging across days. The high and low regimes are determined by selecting the top and bottom 10th-percentile of recorded speeds within a day, as well as the synchronized neural data for predictions. (D) Kullback–Leibler divergence (Kl-div) was calculated between scaled velocity distributions of hand control and decoder predictions, then averaged across folds and days, providing a measure of the fit or ‘distance’ between the two.

To investigate how nonlinear decoders outperform linear approaches, we trained examples of both on four days of offline random target acquisitions from Monkey N and four days from Monkey W. We used RR as the representative linear decoder and three nonlinear decoders: a TCN developed by Willsey *et al* in 2022, a DS movement/posture decoder developed by Sachs *et al* in 2016, and a LSTM recurrent neural network, using the parameters and training from Costello *et al* in 2023 [34, 38, 39]. Within each day, we trained five instances of each decoder and tested them offline on a holdout set, for a total of 20 instances per decoder, per monkey, over time (160 total). Figure 2(A) shows example predictions of Monkey N’s index finger velocities for each decoder, and figure S1(A) shows the same for Monkey W. We measured the average MSE of all models of each type across all days, shown in figure 2(B) for Monkey N and figure S1(B) for Monkey W. In Monkey N, all nonlinear decoders significantly outperformed RR, with respective MSEs 29.1% (TCN), 34.6% (LSTM), and 5.7% (DS) lower (*p* < .01, paired *t*-test) than RR on average, confirming our previous observations [38, 39]. We observed similar but smaller improvements in Monkey W with all nonlinear decoders significantly outperforming RR across all days, with MSEs 8.4% (TCN), 10.0% (LSTM), and 4.5% (DS) lower (*p* < .01, paired *t*-test) than RR on average. Overall, all nonlinear decoders significantly improved over the linear RR decoders, but the ANN-based decoders showed better absolute improvements in Monkey N than Monkey W.

The nonlinear decoders improved over RR in two clear ways: reaching higher speeds during peak movement and maintaining lower speeds during holding periods. To visualize these observations, we produced histograms of the velocity predictions for each model within each day. For Monkey N, single example histograms for each decoder type (all velocities from the test set on one day) are shown in figure 2(C), color-coded by decoder. The filled gray histograms show the true velocities produced by HC. The same histograms for Monkey W can be seen in figure S1(C). We noted that the nonlinear histograms had longer tails and taller tighter peaks than the RR histogram, indicating they produced wider ranges of velocities than RR, but also produced higher proportions of low velocities. Additionally, these differences more closely resembled the histogram produced by HC. To quantify these differences, we compared mean speed and average MSE of nonlinear decoder predictions to RR in the top and bottom 10th percentiles of recorded movements, referred to as the high- and low-speed regimes of movement, across all days. The relative difference in MSE and mean speed between RR and each nonlinear decoder across days can be seen in table 2.

**Table 2.** Relative differences in mean speed and average MSE in different speed regimes, relative to RR. Here, we present the relative difference in mean speeds between each nonlinear decoder and RR in two regimes, the highest 10th-percentile of recorded speeds (per session) and the lowest 10th-percentile of recorded speeds (per session), known as the high and low-speed regimes respectively. Additionally, we present the relative difference in MSE w.r.t. RR in the second column for each regime. Entries marked with an asterisk (*) indicate significant differences (one-tailed, indicated by the column label, *p* < .01) between the listed decoder and RR.

		High-speed regime		Low-speed regime	
Monkey	Decoder	Mean speed (% faster than RR)	Average MSE (% lower than RR)	Mean speed (% slower)	Average MSE (% lower)
N	DS	28.3%*	16.5%*	38.5%*	17.2%*
	TCN	58.9%*	36.6%*	64.0%*	72.6%*
	LSTM	74.3%*	43.1%*	59.8%*	70.1%*

W	DS	25.3%*	11.5%*	27.5%*	−6.6%
	TCN	33.7%*	17.2%*	26.3%*	−11.8%
	LSTM	41.0%*	19.5%*	18.9%*	−16.7%

For Monkey N, the mean speeds of nonlinear decoder predictions in the high-speed regime were significantly faster than mean RR predictions (*p* < .01, paired *t*-test). This increase in speed corresponded with significantly lower average MSEs than RR in the high-speed regime (*p* < .01, paired *t*-test) for all three decoders. We observed a similar but less pronounced effect in Monkey W, whose behavior was less consistent. The significantly faster mean speeds in the high-speed regime (*p* < .01, paired *t*-test) corresponded to significantly lower MSE in the regime for all three nonlinear decoders relative to RR. This was not at the cost of worse performance in the low-speed regime, however. In that regime, mean speeds of nonlinear decoder predictions for Monkey N were significantly slower than mean RR predictions (*p* < .01, paired *t*-test). This resulted in a lower average MSE than RR in the low-speed regime (*p* < .01, paired *t*-test). We observed the same decrease in mean speeds in the low-speed regime for Monkey W (*p* < .01, paired *t*-test), although it did not correspond with a reduction in MSE, but a slight increase. Overall, these results suggest that nonlinear decoders can move significantly faster and slower than the linear RR model, which subsequently improves performance offline.

Next, we investigated how the ‘distribution’ of velocities produced by each decoder compares to the distribution produced by native HC. To do so, we calculated the KL-divergence between true HC histograms and decoder histograms (both scaled to estimate PMFs) each day, as shown in figures 2(d) and S1(D) for Monkeys N and W respectively. We then compared the average divergences of the nonlinear decoders to the divergence of RR decoders across all sessions. In both monkeys, nonlinear decoders showed significantly lower divergences (paired *t*-test, *p* < .01) from HC than RR. Respective divergences 77.7% (DS), 92.4% (TCN), and 89.2% (LSTM) lower than RR for Monkey N and 85.9% (DS), 75.8% (TCN), and 63.2% (LSTM) lower than RR (paired *t*-test, *p* < 0.01) for Monkey W. These results show that the nonlinear decoders can more closely match the ‘true distribution’ of recorded velocities than RR, ultimately improving overall performance. This effect was most pronounced for the ANNs in Monkey N and for the DS decoder in Monkey W.

### 3.2. ReFIT-TCN improves fit to true distribution during real-time control

**Figure 3.**
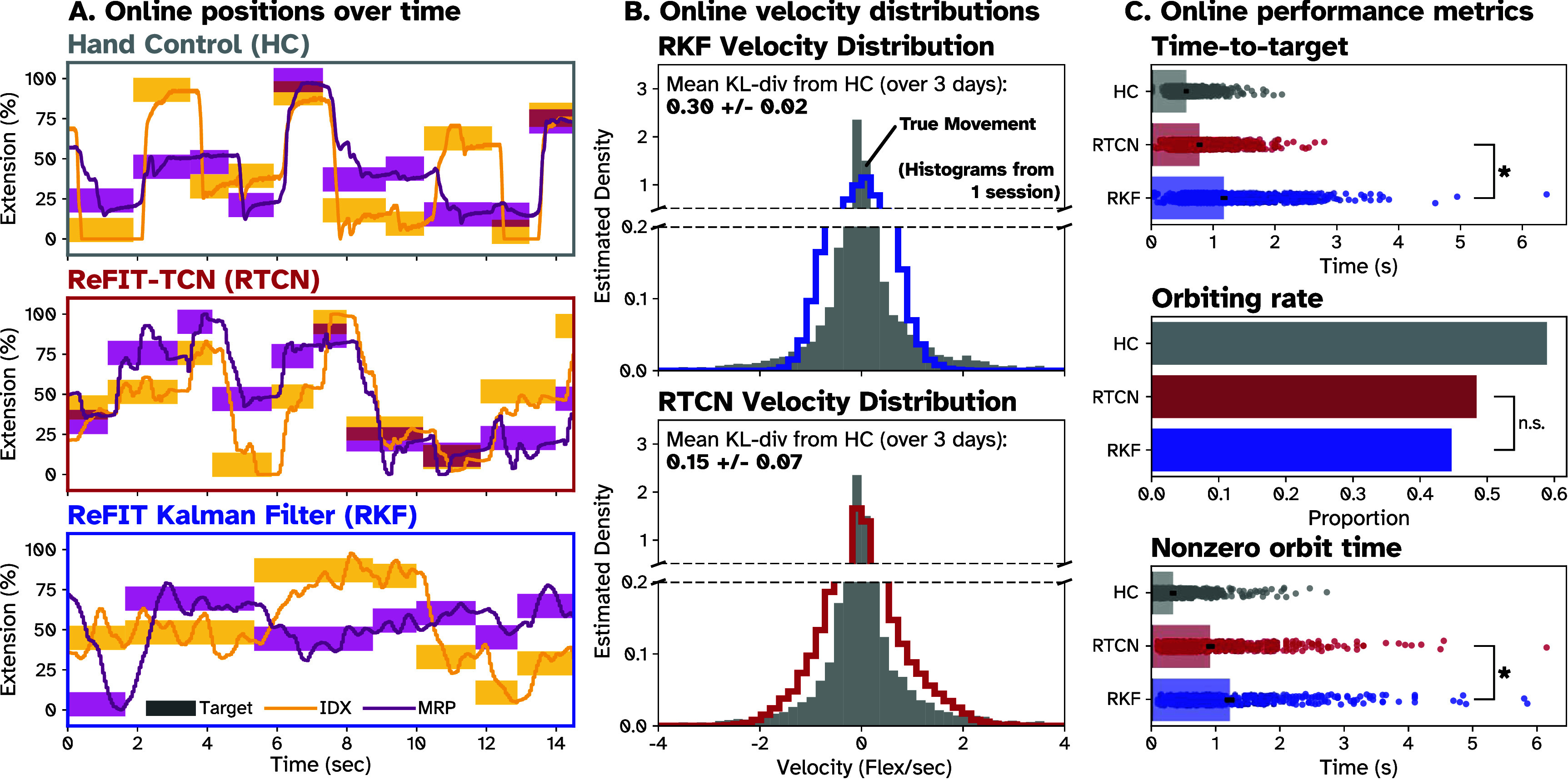
Comparing decoder predictions to hand control movements in real-time control in Monkey N. (A) Position of the virtual hand across sample trials during HC, RKF control, and RTCN control. Finger groups are differentiated by color (IDX yellow, MRS purple). Units are in % extension, covering the full movement range. (B) Example histograms showing the distribution of velocities produced by actual hand movements and real-time predictions on one day. The gray histograms show a representative distribution of true velocities using all hand-controlled training trials for the day. The blue and red histograms show the distributions of all RKF and RTCN trials for the day, respectively. Mean and SD of KL-divergence from the HC histogram across days for each decoder is also shown. (C) Per-trial online performance metrics evaluating the abilities of each decoder to reach and stop in the target. Each point is a single trial, with the bars showing the mean across all trials and all days. From top to bottom: Mean time-to-target (excluding trials with zero TT), orbiting rates, and nonzero orbiting time for HC (gray), RTCN (red), and RKF (blue). Error bars denote standard error (across trials and days). Asterisks (*) indicate RTCN has significantly lower (*p* < .01, one-tailed two-sample *t*-test) average time-to-target and nonzero orbiting time than RKF. N.S. indicates no significant difference found in a one-tailed difference of proportions test for orbiting rate between RKF and RTCN across all trials on all days.

While we have previously demonstrated that ReFIT-TCN (RTCN) outperforms the linear RKF online [38], it is unclear if this is due to the same improvements observed offline in figure 2. To find out, we compared RTCN and RKF on three days with Monkey N, RTCN and TCN on two days with Monkey W, and RKF and TCN on one day for Monkey W. Example position traces for HC, RKF, and RTCN are shown in figures 3(A) (Monkey N) and figure (Monkey W), with the index (IDX) position in yellow and the MRS position in purple. Across all days, Monkey N had success rates of 99.7%, 99.6%, and 91.6% for HC, RTCN, and RKF trials respectively. Across all days, Monkey W had success rates of 83.6%, 90.1%, 56.5%, and 73.2% for HC, RTCN, RKF, and TCN respectively. The following analyses were performed only on successful trials for each decoder.

During experiments, we observed that RTCN appeared to better resemble the high acceleration ‘snappy’ movements produced by true HC than RKF, suggesting RTCN may exhibit the same improvements to the distribution of velocities during real-time control as seen offline. To confirm this, we produced velocity histograms across all RKF (blue), RTCN (red), TCN (purple, Monkey W only) and HC (gray) within each day. Example histograms for one day are shown in figures 3(B) and S2(B) for Monkey N and Monkey W respectively. Across days, the average KL-divergence of RTCN from HC was 51.2% and 58.5% lower (Monkey N and Monkey W, respectively) than the average RKF KL-divergence from HC. Additionally, in Monkey W, average KL-divergence of RTCN predictions was 50.0% lower than TCN (TCN was 17.0% lower than RKF). Per-day KL-divergence can be seen in table 3, along with trial counts. These results suggest that online, RTCN and TCN produce a distribution of movements closer to HC than RKF, like what we observed offline between TCN and RR, although with a relatively smaller improvement.

**Table 3.** Real-time performance comparison between RTCN and RKF on three days. Mean Time-to-target and nonzero orbiting time are included along with the respective SD and number of trials per decoder within each day. Orbiting rate (OR) and KL-divergence from hand control are also reported with the total number of trials included for each decoder on each day. Since overall KL-divergence is measured by an unweighted average of the measurements over days, the # trials is not included.

			Time-to-target, ms			Nonzero orbit time, ms			Orbiting rate		KL-Div from HC	
Monkey:	Performance metrics:		Mean	SD	*# trials*	Mean	SD	*# trials*	Rate	*# trials*	Div	*# trials*
N	Day 1	RKF	1355	737	*373*	1563	1107	*193*	0.51	*382*	0.29	*382*
		RTCN	739	399	*437*	1173	935	*270*	0.60	*451*	0.16	*451*
	Day 2	RKF	1298	702	*362*	1212	835	*133*	0.36	*372*	0.32	*372*
		RTCN	843	391	*476*	787	512	*192*	0.39	*489*	0.21	*489*
	Day 3	RKF	864	330	*375*	857	581	*183*	0.48	*385*	0.29	*385*
		RTCN	742	317	*381*	664	458	*181*	0.47	*388*	0.07	*388*
	Overall	RKF	1171	654	*1294*	1217	926	*509*	0.45	*1139*	0.30	N/A
		RTCN	778	377	*1110*	914	744	*643*	0.48	*1328*	0.15	N/A

W	Day 1	TCN	1718	1034	*184*	1593	1391	*108*	0.57	*190*	0.25	*190*
		RTCN	1423	943	*433*	1538	1252	*237*	0.53	*448*	0.09	*448*
	Day 2	RKF	2512	1432	*165*	1741	1296	*77*	0.45	*170*	0.26	*170*
		TCN	1668	1132	*103*	1633	1299	*69*	0.64	*107*	0.17	*107*
	Day 3	TCN	1991	1123	*90*	2268	2012	*62*	0.67	*93*	0.25	*93*
		RTCN	1599	1118	*49*	1342	885	*33*	0.65	*51*	0.13	*51*
	Overall	RKF	2512	1432	*165*	1741	1296	*77*	0.45	*170*	0.26	N/A
		TCN	1770	1087	*377*	1790	1573	*239*	0.61	*390*	0.22	N/A
		RTCN	1441	962	*482*	1514	1214	*270*	0.54	*499*	0.11	N/A

From the examples in figures 3(A) and S2(A), we observed that RTCN and TCN provided two distinct advantages over RKF: First, RTCN/TCN appeared to reach targets more quickly than RKF. Second, RTCN/TCN appeared to settle into targets more quickly once reached. To investigate the first advantage, we measured mean TT for all HC, RTCN, TCN (for Monkey W) and RKF trials across all days, as shown in figures 3(C) and S2(C). Each dot represents the TT for a single trial, while the mean is represented by the bar, either across days for Monkey N or within a day for Monkey W. Per-day means, standard deviations, and trial counts are listed in table 3. Across days, mean TT using RTCN was 33.6% and 42.6% faster than using RKF (one-tailed two-sample *t*-test, *p* < .01) for Monkeys N and W, respectively. Additionally, across all Monkey W sessions, mean TT using TCN was 29.6% faster than using RKF (one-tailed two-sample *t*-test, *p* < .01). This lower TT suggests RTCN/TCN reached targets more quickly than RKF, outperforming RKF in the high-speed regime, maintaining the improvements seen in offline analyses.

To measure how quickly decoders settled into targets, we first measured OR for all successful HC, RTCN, and RKF trials across all sessions for both monkeys, shown in figure 3(C) (Monkey N) and figure S2(C) (Monkey W). We used a difference of proportions test to compare the ORs of RTCN (in both monkeys) and TCN (in Monkey W) to RKF across all sessions. RTCN had a higher OR than RKF, but not significantly (*p* = 0.03/*p* = 0.02 for Monkeys N and W, respectively). TCN, however, had significantly higher ORs than RKF (*p* < .01). Importantly, HC had much higher ORs across monkeys and sessions than any decoder. We then compared the mean nonzero OT to measure how quickly decoders settled if they did overshoot. Across all Monkey N sessions, RTCN trials averaged a 24.9% lower nonzero OT than RKF (*p* < .01, two-tailed, two-sample *t*-test). In Monkey W, however, average OT for RTCN and TCN were 13.0% lower and 2.2% higher than RKF (n.s., *p* > .01, two-tailed, two-sample *t*-test). These results suggest that RTCN/TCN orbit at similar rates to RKF, if not slightly more. In both monkeys, RTCN makes up for this by correcting OTs more quickly than RKF, although to a reduced extent in Monkey W. Altogether, the results of these real-time comparisons agree with our observations offline. In both monkeys, but to a lesser extent in Monkey W, nonlinear decoders like RTCN/TCN produce a distribution of velocities which more closely matches the true distribution of HC velocities. This enables both monkeys to move faster and reach targets more quickly using RTCN/TCN than RKF and enables Monkey N to correct said overshoots more easily than RKF, echoing the fast/slow regime improvements seen offline.

### 3.3. Modern regularization techniques improve consistency of TCN convergence

**Figure 4.**
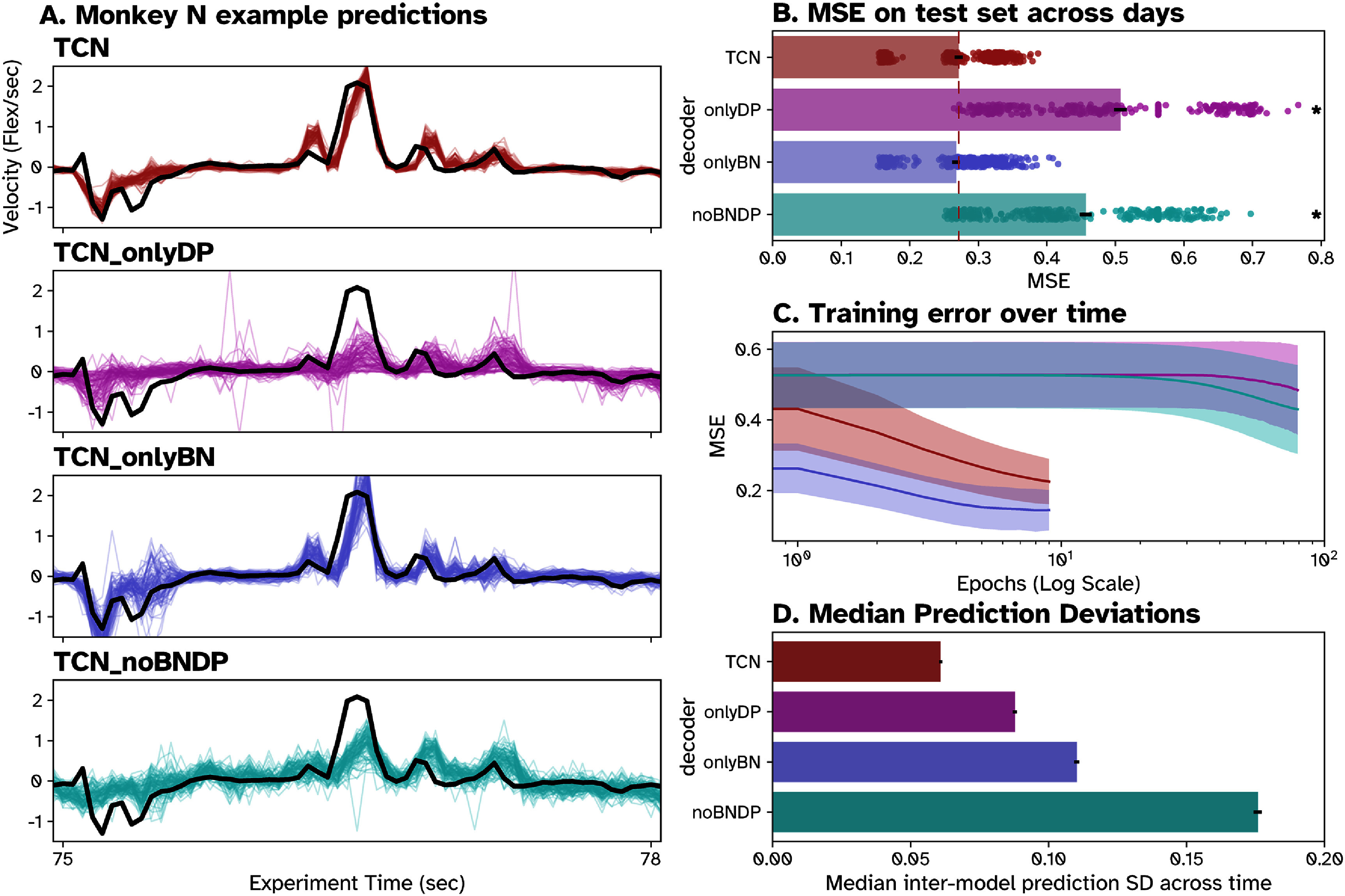
Contribution of different ML regularization techniques to TCN performance in Monkey N. (A) Example predictions on hold-out data from 100 models each of TCN (red), TCN with neuron dropout only (TCN_onlyDP, pink), TCN with batch normalization only (TCN_onlyBN, blue) and TCN with neither (TCN_noBNDP, turquoise) models trained on identical data compared to true hand velocities (black). (B) Bar plot showing mean MSE across all models of each type over all days, with standard error about the mean as black bars. Individual model performances are marked with dots (*n* = 400). Asterisk (*) indicates significantly (*p* < .01) higher average MSE than TCN.(C) Semilog plot of MSE on the training set at each training epoch for each model type. The solid line shows the average training error across all instances on all days for each type, while the shaded area shows the standard deviation. (D) Average prediction deviation across all predictions on all days for each decoder type. Median prediction deviation was measured by first taking the standard deviation across all models of one type at each timepoint in the test prediction, then taking the median across all time points on all days.

While ANNs improve decoding throughput, stochasticity in their training can lead to issues with convergence and unpredictable (potentially hazardous) outputs. We sought to evaluate how well two regularization strategies, BN and neuron DP, address these issues during TCN training. On four days per monkey, we trained 100 models each of TCN and three variants (TCN_onlyDP, TCN_onlyBN, and TCN_noBNDP) on identical training data and tested them on a holdout set. Example predictions for all 100 instances of each variant can be seen in figures 4(A) (Monkey N) and S3(A) (Monkey W). In table 4 (and figures 4(B), S3(B) we compared the average MSE of ablated models and TCN across days. In both monkeys, both the onlyDP and noBNDP models performed significantly worse than the standard TCN (one-tailed, two-sample *t*-test, *p* < .01), as shown in the first column of table 4. The onlyBN model performed significantly worse than TCN in Monkey W, but not Monkey N. Notably, the onlyBN and TCN models achieved higher performance in 10 epochs than onlyDP and noBNDP in 80 (figures 4(C) and S3(C)). These results suggest batchnorm contributes heavily to improving model performance and training speed, whereas neuron DP does not. Figures 4(A) and S3(A), appear to show that while neuron DP did not improve performance or training speed, but did lead to more consistent model convergence. To measure this, we calculated the median prediction deviation across all instances of each model across days, shown in table 4. This confirmed our observations, as onlyBN and noBNDP models had 81.1% and 188.9% higher deviations than TCN for Monkey N and 145.7% and 123.8% higher deviations than TCN for Monkey W. These findings suggest that neuron DP is the main contributor to consistent model convergence over BN. Overall, we found that batchnorm and neuron DP allow TCN to train quickly, reach high performance, and consistently converge on the same solution.

**Table 4.** Average MSEs and Median Prediction Deviations for TCN and three ablated model variants. The columns labeled ‘MSE’ and ‘(N) MSE’, contain the average (and std. deviation) of prediction MSE across 400 models and 4 days for each of the four variants of TCN tested. An asterisk (*) denotes a significantly higher average MSE than TCN (*p* < .01, one-tailed *t*-test). The second two data columns report the median prediction deviations across the same groupings. In the columns marked with (N), models were trained on normalized training data to test the effects of normalization of the entire dataset over batch normalization. The bold numbers in the MSE columns show the lowest MSE for each monkey.

Monkey	Model variant	MSE	(N) MSE	Median deviation	(N) Median deviation
N	TCN	0.27 + 0.07	0.27 + 0.07	0.061	0.063
	TCN_onlyDP	0.51 + 0.13*	0.31 + 0.09*	0.088	0.052
	TCN_onlyBN	**0.27 + 0.06**	0.27 + 0.06	0.110	0.146
	TCN_noBNDP	0.46 + 0.12*	**0.25 + 0.06**	0.176	0.076

W	TCN	**0.38 + 0.08**	**0.37 + 0.08**	0.077	0.067
	TCN_onlyDP	0.50 + 0.13*	0.41 + 0.09*	0.085	0.087
	TCN_onlyBN	0.43 + 0.09*	0.46 + 0.09*	0.189	0.265
	TCN_noBNDP	0.49 + 0.12*	0.43 + 0.08*	0.172	0.177

We repeated the same analyses but normalized neural and behavioral data, training all models for 15 epochs (figures S4(A) and S5(A) for Monkey N and W respectively). In both monkeys, normalization improved average performance (in MSE) and training time (to 15 epochs) in models with no previous normalization, TCN_onlyDP and TCN_noBNDP figures S4(B)–(C) and S5B–(C). Normalization increased median prediction deviation in the onlyBN model for both monkeys S4(D) and S5(D). Overall, normalization of the entire dataset has similar effects to BN, improving model performance and training times when no other form of normalization was used.

### 3.4. Online stability of TCN

**Figure 5.**
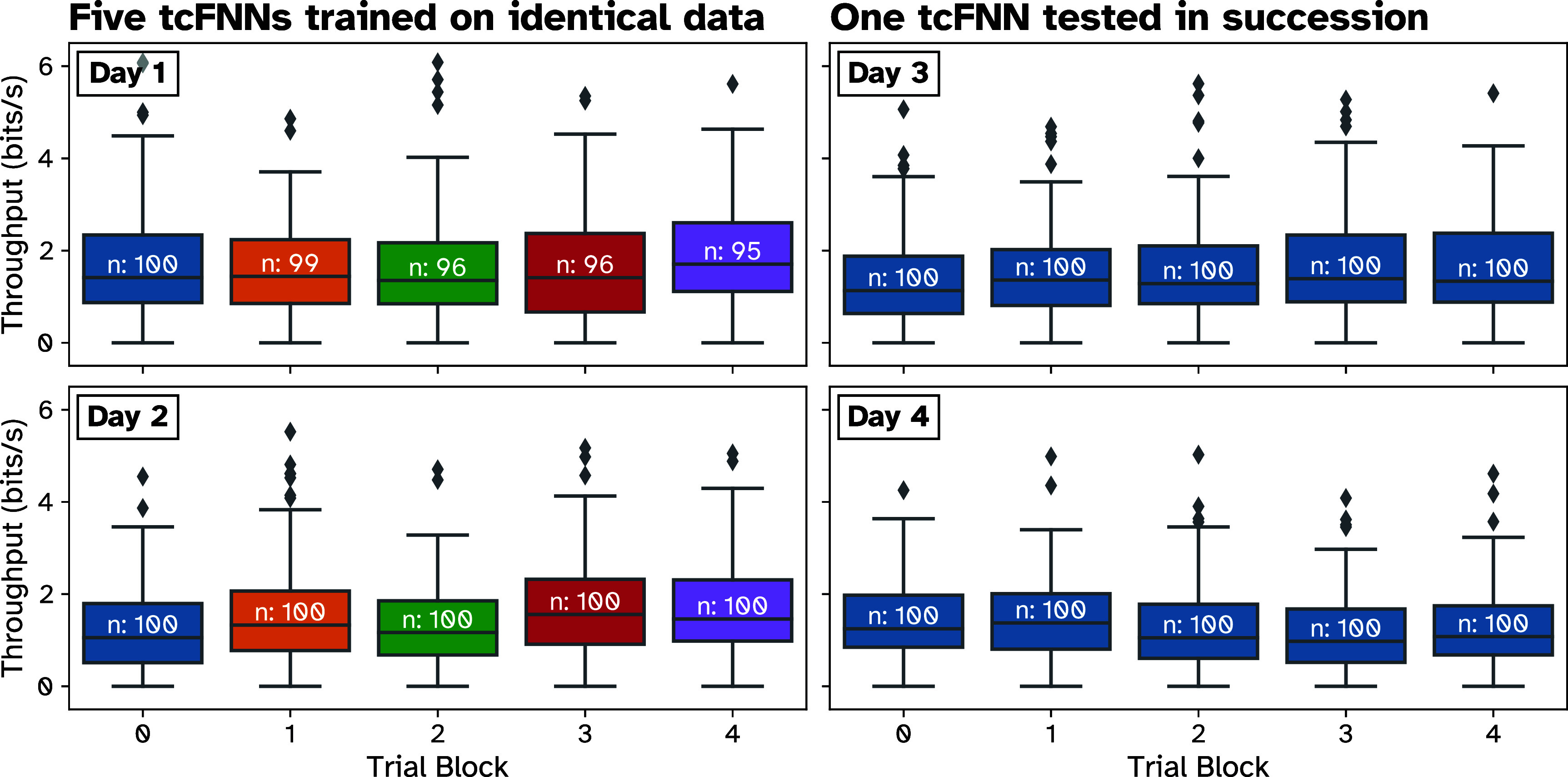
Validating stability of TCN (non-ReFIT) decoders trained on identical training datasets in real-time control. Color indicates a different decoder tested (within each day) Left Column: on 2 d (1 per plot), five TCN decoders were trained on identical training data (within a day) and tested in succession. Per-trial throughput was measured in bits s^−1^. Each set of box plots shows the throughput median, quartiles, and IQR for each decoder tested (indicated by color) within a day. Right Column: on two separate days, a single TCN was tested six times in succession as a control experiment, simulating decoder switches after ∼100 trials.

Observing that offline consistency is improved by using regularization techniques, we next aimed to evaluate performance consistency during online, closed-loop trials. To assess this, on two days, Monkey N performed successive blocks of trials using five TCNs (without applying ReFIT) trained on identical training data. Additionally, we performed a control on two separate days, where Monkey N performed five consecutive blocks of trials using only one TCN. Throughput (bit rate) was used as a measure of trial performance. Each plot in figure 5 shows box plots summarizing the per-trial throughput of each trial block within each day. Since stochastic initialization of each TCN instance could contribute to variance in decoder convergence, ReFIT recalibration was not applied, so overall performance is lower than observed in previous studies [38]. To test for any major differences in performance between trial blocks in and across days, we performed a nested ANOVA, a hierarchical two-way ANOVA with each session/day as the high-level factor and intra-day trial block as the low-level factor. The nested ANOVA found a significant effect of day (*p* < .01) on average bitrate but found no significant effect (*p* > .01) of intra-day trial block nor a significant interaction (*p* < .01) between day and intra-day trial block. This means that average overall performance changed significantly from day-to-day, which we have observed previously, but that within each day, on average, there was no significant difference between trial blocks. Additionally, the lack of significant interactions between the two factors suggests that there is no significant effect of each specific day on the average between trial blocks. If the days in which different models trained on the same data introduced significantly more variability, we would expect to see some significance either in the intra-day trial block factor or in the interaction. Thus, we concluded that the variance in TCN convergence does not introduce significant changes in real-time performance.

### 3.5. Impact of task changes to offline decoder predictions

**Figure 6.**
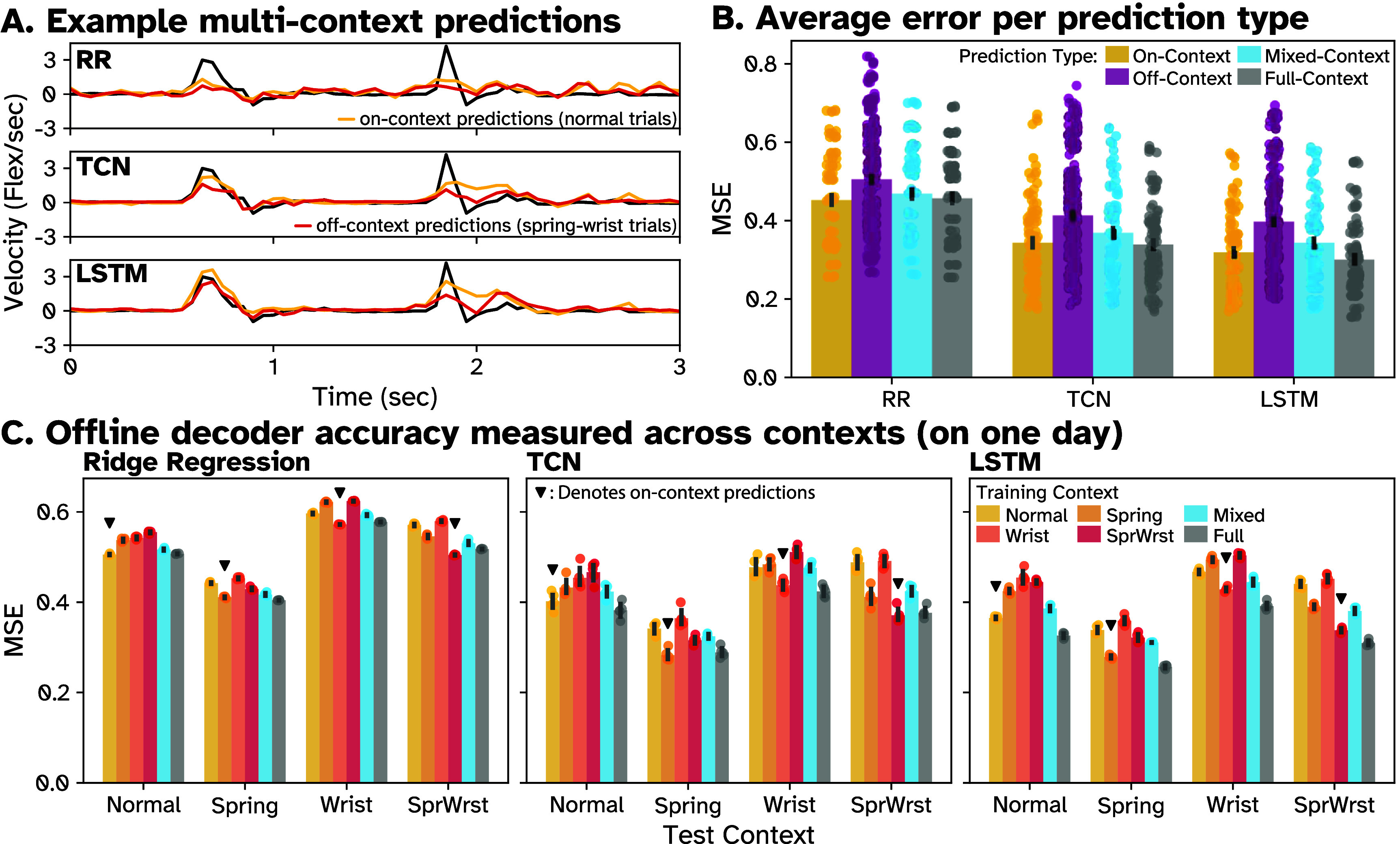
Measuring impact of context shifts on open-loop predictions for RR and TCN decoders across days. On five days, five ∼400 trial training datasets were prepared for Monkey N performing trials in each of four contexts: Normal, Spring, Wrist, and Spring-Wrist. Additionally, ten additional datasets were constructed per day, five ‘mixed’ and five ‘full’, each containing ∼100 trials and ∼400 trials from each of the four contexts, respectively. TCN and RR decoders were then trained on individual datasets and then tested on 100 held-out trials of each single context. (A) Example predictions on normal trials for RR, TCN, and LSTM. In yellow, we see predictions from decoders trained on the same context (‘normal’) as the test data (shown in black). In red, we see predictions from decoders trained on a different context (‘spring-wrist’) than the test data. (B) Average decoder MSE grouped into ‘on-context’, ‘off-context’, ‘mixed-context’, and ‘full-context’ predictions. On-context predictions (yellow) were made by decoders tested on trials from the same context on which they were trained (downward triangle in C). Off-context predictions (purple) refer to predictions made on a different context as a decoder’s training data. Mixed-context predictions (blue) were made by decoders trained on a composite dataset containing data from all contexts but limited to the same size as a single context dataset. Full-context (gray) predictions were made by decoders trained on a dataset comprising four full single context datasets. While bars represent average MSE across all sessions, dots represent the MSE of a prediction on a single test dataset by a single decoder. (C) Average MSE for each combination of decoder training and testing context within one day, across five decoders per bar. Colors represent the source (training) context, and groups represent the target (test) context. Dots represent the MSE of a prediction of a single decoder a single test dataset (five test datasets per context per day). In each group, a downward triangle indicates decoders trained and tested in the same context. Standard error about the mean is shown as black bars.

We next investigated the generalization ability of the two ANN decoders, TCN and LSTM, to task context changes, similar to a previous study which looked at linear decoders [32]. To evaluate this, we collected 600+ trials per day with Monkey N of four contexts on five days—normal, spring, wrist, and spring-wrist, and split them into training (400 trials per fold) and testing datasets (100 trials held out), as described in the Methods. Within each day, we trained TCN, LSTM, and RR models on each training set (including mixed datasets with data from multiple contexts) and tested them on four offline single-context holdout datasets. In figure 6(A), we show examples predictions from each model type on test data from the ‘normal’ context. In yellow, we can see predictions from a decoder trained on normal trials (on-context). In red, we plot predictions from a decoder trained on spring-wrist trials (off-context), which appear worse than the on-context prediction.

Figure 6(C) shows the mean MSEs of the ANN and RR decoders trained and tested on each combination of contexts on a single day. Across all combinations on all days (six training contexts, four test contexts, five predictions per combination of train and test context, and 5 d, for a total of 600 predictions per decoder type), the ANN decoders significantly outperformed RR, with respective MSEs 25.5% (LSTM) and 20.8% (TCN) lower than RR (paired *t*-test, *p* < .01). Average LSTM MSE was 5.9% lower than TCN respectively (*p* < .01). Qualitatively, we observed that all decoders types had the lowest MSEs when tested *‘on-context’* (the same training and testing context), as seen by the downward arrows in figure 6(C). Performances were grouped by ‘on-context’ and ‘off-context’ predictions across all days and averaged, shown in figure 6(B). Both RR and ANN decoders performed significantly worse off-context than on-context, with average off-context MSEs 11.7%, 20.4%, and 24.6% higher than on-context for RR, TCN, and LSTM decoders respectively (*p* < .01). These results suggest that all decoders exhibit some overfitting to the training context, but ANN decoders overfit more than RR. However, average off-context MSE for the nonlinear decoders was lower than average on-context RR MSE (one-tailed *t*-test, *p* < .01), suggesting that, in this case, the ability of ANNs to learn more about the task than RR makes up for increased overfitting.

An initial approach to resolving this overfitting problem is to relax the constraints of generalization and include multiple contexts in the training data. To this end, we trained decoders on ‘mixed’ context datasets, which contained the same amount of data as single context datasets but included data from all four contexts. Figure 6(C) shows mixed-context performance on a single day of examples and figure 6(B) shows the average performance across all mixed decoders on all days. Across all days, mixed-context decoders performed significantly better than off-context decoders (*p* < .01), achieving average MSEs 7.4%, 10.7%, and 13.6% lower than off-context decoders for RR, TCN, and LSTM respectively. Average mixed-context MSE was 3.5%, 7.4%, and 7.7% higher (n.s., *p* > .01) than on-context MSE for RR, TCN, and LSTM respectively. While all decoders showed significant improvements when trained on richer datasets, the ANN decoders improved by a larger margin. We then trained decoders on ‘full’ datasets containing four entire single-context datasets (e.g. 1600 trials instead of 400). These slightly but significantly outperformed the mixed-context decoders (*p* < .01) and achieved similar performance to on-context decoders. On average, full-context ANN decoders outperformed on-context decoders (1.3% and 5.7% for TCN and LSTM, respectively). Full-context RR decoders achieved average MSEs 1.0% higher than on-context. These results show that while all decoders benefit from richer and larger datasets, ANNs showed better relative improvement, suggesting they more efficiently leverage the new data.

## 4. Discussion

Nonlinear decoders, especially ANNs, are increasingly used in movement and speech BCIs for their improved performance [4, 8, 35–37, 48], though, the reasons behind their performance gains are not well understood. We found that TCN and other nonlinear decoders improve performance by better matching the able-bodied velocity distributions during decoding. We also highlighted the role of regularization techniques, namely batchnorm and DP, in ensuring reliable, high performance ANN decoders. Finally, we showed how ANN-based decoders can leverage richer datasets to improve performance across multiple task contexts. Overall, these findings provide insight into how to develop and implement neural networks for high-performance BCI applications. This study has several key limitations: We use data from only two NHP subjects performing a constrained 2-DOF task, one of which (Monkey W) often struggled to perform the task. Our findings may not generalize to other tasks or subjects. Only three types of nonlinear decoders were tested, and no hyperparameter tuning was performed on the ANNs, instead using the values provided by previous studies [38, 39]. Future work should explore less constrained tasks using a broader set of decoders in more subjects.

While nonlinear BMI decoders are known to outperform linear methods, our work suggests that they also better mirror true HC [33–35, 38, 39]. As seen in figure 2 and S1, linear decoders tend to produce Gaussian distributions of velocities, which, combined with the dynamics of the Kalman Filter, limits decoders to a ‘slow and steady’ control strategy. This produces smooth movements but ultimately leads to slower real-time control. Nonlinear decoders, on the other hand, are not limited to this control strategy. Anecdotally, we observed that the nonlinear decoders tested online better resembled true HC and allowed the monkeys to adopt their preferred control strategy more easily, which was to quickly move to the general area of the target and make quick corrective movements as needed to finally settle in the target. We believe the results of this study confirm this observation. By better replicating user intention/control strategy, these decoders may facilitate embodiment of the BMI, an issue that has been shown to contribute heavily to device abandonment for devices like upper-limb prosthetics [49]. However, cross-subject generalization is an equally relevant concern for real-world deployment of BMIs [50], so future studies should include a more thorough investigation into the impact of inter-subject behavioral differences on the ability of nonlinear decoders to better replicate true HC.

While ANN-based decoders have been proposed for BMIs since the early 2010’s, they did not take off until the last 5 years or so. This might be due to the fact many of the most common approaches for network regularization and performance improvement (Adam optimization, neuron DP, BN, ReLU) were published between 2014–2016 and took time to gain traction in the field [46, 47, 51, 52]. Before this point, inconsistent convergence was a common nuisance in offline applications of deep learning, and a potential safety hazard when it comes to BMI decoding. Further investigation using comparative techniques like projection-weighted canonical correlation analysis should be performed to check if the internal structure of these networks converges as consistently as their outputs [53].

In the context-shifting task, the ANN decoders exhibited worse overfitting than RR, as might be expected. However, even in a challenging generalization task (single context to multiple), off-context ANNs still outperformed on-context RRs. By relaxing this constraint and training on multiple contexts, both TCN and LSTM predictions matched and, in some cases, slightly surpassed on-context decoder performance. As BCI training moves towards using more behavior-rich and free-behaving datasets, ANNs are well-suited to take advantage of the richer information, especially with data augmentation strategies like noise injection [54]. It should be noted that the overall performance on multiple seen contexts is markedly different than generalization to unseen domains. However, more advanced transfer and continual learning approaches aim to enable ANN-based decoders to generalize by transferring knowledge between tasks [54–57]. Two recent studies have shown that incorporating transfer learning into decoder training improves stability over time and allows for cross-species decoding [50, 58].

In this work, we explored ‘*how*’ nonlinear decoders improve over linear methods but did not address underlying reasons ‘*why*’. While some research suggests that individual neural firing rates are linearly correlated with behavior [22, 24], many others also support a highly nonlinear relationship between behavior and neural activity in the motor cortex [25–28, 59]. Additionally, spinal interneurons in the motor pathway provide places for nonlinear integration of information end effector [60, 61]. This is not to say that the TCN model is a valid computational model of corticomotor projections or motor control, as it omits key components like recurrence and input from other brain areas [62–65]. A thorough investigation into the internal structure of ANN-based decoders with respect to the structure of the corticospinal motor pathways may uncover if these decoders are more biologically relevant than linear approaches.

## Data Availability

The data and code that support the findings of this study are openly available at the following URL/DOI: https://chestekresearch.engin.umich.edu/data-and-resources/.
